# Protocol and biomarker strategy for a multi-site randomized controlled trial examining biological mechanisms and dosing of active music engagement in children with acute lymphoblastic leukemia and lymphoma and parents

**DOI:** 10.1186/s12906-023-03909-w

**Published:** 2023-03-27

**Authors:** Sheri L. Robb, Kristen A. Russ, Steven J. Holochwost, Kristin Stegenga, Susan M. Perkins, Seethal A. Jacob, Amanda K. Henley, Jessica A. MacLean

**Affiliations:** 1 School of Nursing, Indiana University, 600 Barnhill Drive, Indianapolis, IN 46202 USA; 2 School of Medicine, Indiana University , 351 W 10th Street, Indianapolis, IN 46202 USA; 3 Department of Psychology, Lehman College, City University of New York, 250 Bedford Park Boulevard, West Bronx, NY 10468 USA; 4 Children’s Mercy Hospital, 2401 Gillham Road, Kansas City, MO 64108 USA; 5 School of Medicine, Indiana University, 340 W 10th Street, Indianapolis, IN 46202 USA; 6 School of Medicine, Indiana University, 410 W 10th Street, Indianapolis, IN 46202 USA; 7 Purdue School of Engineering and Technology, IUPUI, 535 W. Michigan Street, Indianapolis, IN 46202 USA

**Keywords:** Music therapy, Biomarker, Acute lymphoblastic leukemia, Study protocol, Stress, Immune function

## Abstract

**Background:**

Music therapy is a standard palliative care service in many pediatric and adult hospitals; however, most research has focused on the use of music to improve psychosocial dimensions of health, without considering biological dimensions. This study builds on prior work examining psychosocial mechanisms of action underlying an Active Music Engagement (AME) intervention, designed to help manage emotional distress and improve positive health outcomes in young children with cancer and parents (caregivers), by examining its effects on biomarkers of stress and immune function.

**Methods:**

This two-group randomized controlled trial (R01NR019190) is designed to examine biological mechanisms of effect and dose-response relationships of AME on child/parent stress during the consolidation phase of Acute B- or T-cell Lymphoblastic Leukemia (ALL) and T-cell Lymphoblastic Lymphoma (TLyLy) treatment. Child/parent dyads (n = 228) are stratified (by age, site, risk level) and randomized in blocks of four to the AME or attention control condition. Each group receives one session (30-minutes AME; 20-minutes control) during weekly clinic visits (4 weeks standard risk B-cell ALL; 8 weeks high risk B-cell ALL/T-cell ALL/TLyLy). Parents complete questionnaires at baseline and post-intervention. Child/parent salivary cortisol samples are taken pre- and post-session (sessions 1–4). Child blood samples are reserved from routine draws before sessions 1 and 4 (all participants) and session 8 (high risk participants). We will use linear mixed models to estimate AME’s effect on child/parent cortisol. Examining child/parent cortisol as mediators of AME effects on child and parent outcomes will be performed in an ANCOVA setting, fitting the appropriate mediation models using MPlus and then testing indirect effects using the percentile bootstrap approach. Graphical plots and non-linear repeated measures models will be used to examine dose-response relationship of AME on child/parent cortisol.

**Discussion:**

During pediatric cancer treatment there are special challenges that must be considered when measuring cortisol and immune function. In this manuscript we discuss how we addressed three specific challenges through our trial design. Findings from this trial will increase mechanistic understanding of the effects of active music interventions on multiple biomarkers and understanding of dose-response effects, with direct implications for clinical practice.

**Trial Registration:**

ClinicalTrials.gov: NCT04400071.

**Supplementary Information:**

The online version contains supplementary material available at 10.1186/s12906-023-03909-w.

## Background

Music therapy has become a standard palliative care service in many pediatric and adult hospitals in the United States [1]. According to a survey of Children’s Hospital Association members, about 70% of the 245 hospitals surveyed reported offering music therapy services to their patients and families [2, 3]. Cancer treatment is an inherently stressful experience for both young children and their parents and their outcomes are interrelated [4–7]. Both children and parents experience emotional distress and poor quality of life, and many parents experience traumatic distress symptoms because of their child’s cancer diagnosis and treatment [8–12].

Although positive outcomes resulting from psychosocial interventions have been associated with improvements in biological function including decreased cortisol production and improved immune function [13–19], most music therapy research has focused on psychosocial dimensions of the cancer treatment experience, rather than its biological dimensions [20–22]. Coupled with growing evidence that active music experiences affect neuroendocrine and immune responses in other patient populations [23–29], an examination of active music to diminish emotional distress and improve positive health outcomes in children with cancer and parents is well justified and will address important gaps in our scientific knowledge about the use of music to improve health [30, 31].

The Active Music Engagement (AME) intervention uses interactive music play to counteract stressful qualities of the cancer treatment environment to reduce interrelated parent-child emotional distress and improve positive health outcomes during acute cancer treatment [32–34]. To counteract the lack of mechanistic understanding of music therapy approaches, we designed our current trial (R01NR019190) to examine AME’s effects on stress via Hypothalamic-Pituitary-Adrenal Axis (HPA-axis) activity and immune function (immunomodulatory cytokines). Within the context of pediatric cancer treatment there are special challenges that must be considered when measuring cortisol and immune function. In this manuscript we report our specific aims and study protocol. In addition, we identify and discuss three specific design challenges and how these were addressed.

## Specific aims

Purposes of this two-group, randomized, controlled trial are to examine mechanisms of effect and dose-response relationships of AME on child/parent stress over time. Specific aims are to:

### Aim 1

Compare the magnitude of change in child and parent cortisol levels between AME and control during Acute Lymphoblastic Leukemia (ALL) and T-cell Lymphoblastic Lymphoma (TLyLy) treatment.

###  Hypothesis 1.1

Compared to attention control, *children* in the AME group will have greater pre- to post-session percent decreases in cortisol.

###  Hypothesis 1.2

Compared to attention control, *parents* in the AME group will have greater pre-to post-session percent decreases in cortisol.

### Aim 2

Examine cortisol as a mediator of AME effects on child and parent outcomes during ALL and TLyLy treatment.

###  Hypothesis 2.1

Reductions in child and/or parent cortisol will mediate the effect of AME on child immune function.

###  Hypothesis 2.2

Reductions in child and/or parent cortisol will mediate the effect of AME on *child* emotional distress and quality of life.

###  Hypothesis 2.3

Reductions in child and/or parent cortisol will mediate the effect of AME on parent emotional/traumatic distress symptoms and quality of life.

### Aim 3 (Exploratory)

Examine the dose-response relationship of AME on child and parent cortisol during ALL and TLyLy treatment.

###  Hypothesis 3.1

While we make no hypotheses for this exploratory aim, we anticipate that AME effects may increase, decay, or remain constant over the course of treatment.

## Methods

Methods for this trial are grounded in our previously published work including several pilot studies [33–36] and a manuscript that details of our treatment fidelity strategies [37].

### Conceptual framework

**Fig. 1:**
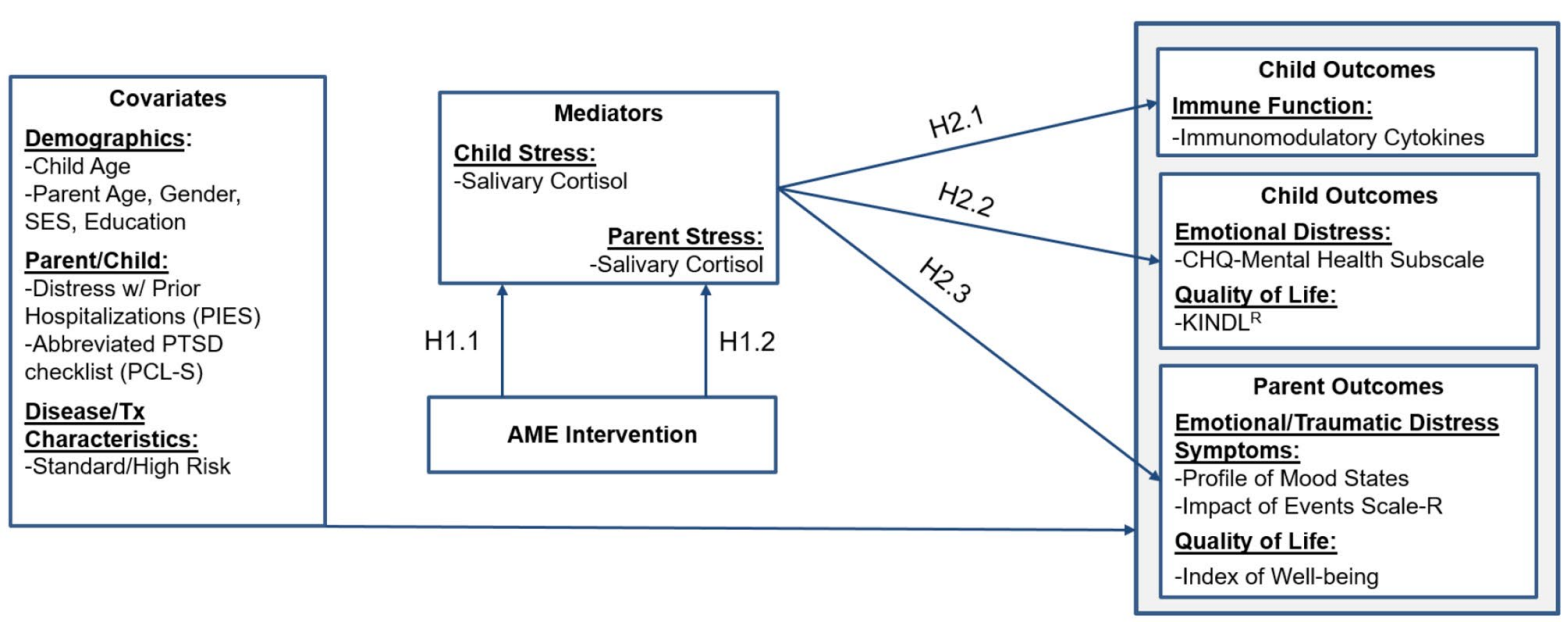
Conceptual Framework

Our conceptual framework (Fig. 1) is based on Robb’s Contextual Support Model of Music Therapy [32, 38], which is grounded in Self-Determination Theory [39] and further informed by Kazak’s Pediatric Medical Traumatic Stress Model [40], which provides a useful heuristic for understanding short and long-term consequences of pediatric cancer treatment for children and their parents. In our conceptual framework, recurring events related to cancer treatment (i.e., clinic visits, procedures) are viewed as stressful, potentially traumatic events.

Parent appraisal of events as traumatic or not traumatic is influenced by pre-existing factors that serve as *covariates* in our study. We based covariate selection on published research that indicates higher parent and child distress during cancer treatment is related to: (1) demographics (younger child/parent age, female parent gender, and lower socio-economic status/education) [41, 42], (2) higher parent/child distress with prior hospitalizations, and greater traumatic stress symptoms [5, 43, 44], and (3) disease and treatment characteristics (treatment intensity) [9–11, 45–49]. In addition, child and parent sex will be important covariates when analyzing our biological data based on evidence that sex hormones play a role in immune response [50, 51] and account for differences in salivary cortisol [52, 53].

We hypothesize that AME directly targets the *mediators* of child and parent biological stress (cortisol). Our study examines child and parent cortisol as mediators of AME intervention effect on *outcomes* for child (immune function, emotional distress, and quality of life) and parent (emotional distress, traumatic distress, quality of life). This study will allow us to determine *how* the AME intervention works at the biological level and the intersection of HPA-axis activity (cortisol) with reductions in emotional distress (emotional/traumatic stress; quality of life) associated with pediatric cancer treatment. Findings will have implications for the use of active music interventions to manage treatment-related distress in other populations.

### Study design and randomization

**Fig. 2:**
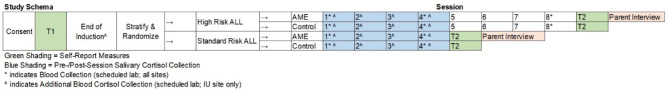
Study Schema

This study is a two group, stratified randomized controlled trial. The trial received Single Institutional Review Board Approval from Indiana University and is recruiting from three children’s hospitals in the United States. Children and one parent (enrolled as dyads) are stratified by child age (preschool 3–5 yrs; school-age 6–8 yrs), site, and treatment intensity (4 weeks standard risk B-cell ALL; 8 weeks high risk B-cell ALL, T-cell ALL, T-cell LyLy), and randomized in blocks of four to the AME intervention or attention control condition (audio-storybooks; ASB). See Fig. 2 for study schema.

Consolidation duration varies by risk level for B-cell ALL participants (4 weeks standard; 8 weeks high). Consolidation duration for all T-cell ALL and T-cell LyLy participants is 8 weeks. Treatment protocols and intensity of treatment are similar for high risk B-cell ALL and T-cell participants so we include all three diagnoses under the term “high risk” when referencing duration and intensity of treatment. As such, standard risk participants receive 4 weekly sessions and high risk participants receive 8 weekly sessions. We are enrolling both standard and high risk participants to examine whether dose-response differs based on treatment duration. It is important to examine changes in child-parent stress over the full treatment cycle, and we will control for variations in number of sessions through stratified randomization and our analytic methods.

Baseline data (Time 1) will be collected after consent and before the start of the consolidation phase of treatment. Time 2 data will be collected immediately following the last study session (and no later than 7 days following last study session). Following Time 2 questionnaires, parents randomized to AME will be offered an optional parent interview (completed within 3 weeks of T2 data collection). Parents/children are encouraged to use AME and low dose play activities between clinic visits and self-reported frequency and duration of activities will be collected during each session. Data collection timelines are based on positive findings from our preliminary studies and inform our primary aims that examine mediation [33, 54]. See Fig. 2 for study schema and Table 2 for assessment timelines.

### Participants

Children and parents (or primary caregiver) will be enrolled as a dyad and must meet the following criteria to be study eligible. *Inclusion criteria*: (1) child is 3–8 years of age at time of enrollment, (2) child has diagnosis of standard or high-risk B- or T-cell ALL or T-cell lymphoblastic lymphoma (TLyLy), (3) child is currently receiving induction therapy, and (4) one parent (≥ 18 years of age) can be present for all sessions. *Exclusion criteria*: (1) child has Philadelphia positive ALL, (2) child has Cushing’s disease, (3) child takes steroid medication for asthma and/or has asthma that is not well-controlled, (4) parent does not speak English, or (5) the child has a significant cognitive impairment that might hinder participation (determination made in consultation with attending physician, oncologist, and parents).

### Sample size and power analysis

We will recruit a total sample size of 228 child/parent dyads and assume 25% attrition to retain 170 child/parent dyads (85 dyads/group) at Time 2. The primary goal of this study is to examine mechanisms via the mediation effects of the AME intervention. We will use the percentile bootstrap method to estimate the indirect/mediated effect [55]. The sample size needed for this study is primarily driven by the power needed for the mediation hypothesis in Aim 2. Simulations of the two-path mediation model in Mplus [56] show that 170 subjects are needed to have 88% power to test the total indirect effect using the Sobel approach when the effect of the independent variable (AME in this case) on the mediator is at least medium (13% of variation explained), mediator on the outcome is between small and medium (7% of variation explained), and independent variable on the outcome is small (2% of variation explained). Via simulation, the Type I error with this sample size under a model that assumes no indirect effect is also less than 0.007, which will readily accommodate the multiple comparison adjustments planned below (e.g. with 7 cytokines a conservative Bonferroni adjustment would be 0.05/7 = 0.007). A sample size of 170 will also afford 90% power to detect a medium effect size for H1.1 and H1.2 based on a two-sample t-test with a two-sided alpha level of 0.05. Fitting models that allow for repeated measures across session will result in improved power. Using the percentile bootstrap method instead of Sobel should afford the same or greater power.

### Setting

Young children (ages 3–8 years) and their parents are being recruited from three children’s hospitals. These hospitals are in metropolitan areas that serve large catchment areas. All three sites are members of the Children’s Oncology Group, and administer chemotherapy according to protocol guidelines, ensuring consistency of treatment across sites. Study conditions and data collection sessions will take place in the outpatient clinic setting.

### Recruitment and informed consent

This study has received single Institutional Review Board Approval from the Indiana University Institutional Review Board which serves a central IRB for all participating institutions. Project managers at each site recruit children/parents during a scheduled clinic visit during the induction phase of treatment. During initial approach, project managers provide initial study introduction/brochure and verify eligibility. For interested families, child/parent dyads are enrolled following written, informed parental consent for parent and child and written assent for children ≥ 7 years, following human subjects review committee requirements.

### Study condition procedures

Children/parents randomly assigned to AME or attention control receive sessions that are similar in length (30 min. AME; 20 min. ASB) and have the same timing of contact (4 weekly sessions standard risk; 8 weekly sessions high risk). In previous AME trials, the AME and ASB conditions were delivered in an in-patient setting and had an equal duration (45 min, with 30 min of music or stories) [33–36]. The current trial takes place in the outpatient clinic setting. To accommodate clinic flow and patient needs we needed to limit session duration to 30 min or less. As such, total session duration for AME sessions is 30 min total (5 min collaborative goal setting; 20 min of music-play; 5 min educational content). Total session duration for ASB is 20 min (5 min set-up; 15 min audio-storybooks – the length of one storybook). Although total session length is not equivalent, the amount of audio-visual stimulation is similar across groups, with the additional 10 min in AME attributed to assessment and educational activities that are unique to that condition. In addition to in-person sessions, participants take home a music-play or audio-storybooks kit to encourage between session use of the condition-related activities [33–37].

Both conditions are standardized (content, materials, and delivery), and all sessions are delivered by a board-certified music therapist in the outpatient clinic setting. All sessions are audio-recorded for quality assurance monitoring to ensure fidelity and prevent provider drift and/or contamination across treatment and control conditions (see Treatment Fidelity below).

#### Active music engagement (AME) intervention

The AME was designed based on the Contextual Support Model of Music Therapy (CSM-MT) [32] which is grounded in Self-Determination Theory [39], and further informed by Kazak’s Pediatric Medical Traumatic Stress Model [40]. The CSM-MT explains how music can be used to create a supportive environment by offering optimal levels of structure, autonomy support, and relationship support [38].

AME intervention sessions were designed for delivery by a board-certified music therapist who tailors music experiences to encourage active engagement in and independent use of music play as a strategy to manage emotional distress. During AME sessions, the music therapist provides children/parents repeated opportunities to experience competence, autonomy, and meaningful interactions through music play activities, which leads to active engagement and positive forms of coping. In addition, the therapist provides supportive education about ways music play can be used to manage distress and promote a sense of normalcy both during and between clinic visits.

There are three components to the AME intervention: (1) therapist-led music play activities, (2) the music play resource kit (to promote independent music play), and (3) tip sheets for parent education and support that focus on information and strategies aimed to help parents use music play to manage their child’s distress (the focus of the AME intervention) during clinic visits, and how to use music play at home. Table 1 shows the relationship of essential AME intervention content to CSM-MT theoretical principles.

**Table 1:** Active Music Engagement Intervention Components and Theoretical Principles

Intervention Component	Theoretical Principles
Component 1: Music-Based Play Activities	1. Predictable environments provide structure that supports child *competence*. Therapist uses familiar music activities to provide structure and increase child’s ability to predict what will happen in their environment. 2. Leveled activities help ensure success and support child *competence*. Therapist tailors physical activity requirements to meet the individual needs of each child. Enables child success and engagement during periods of high or fluctuating symptom distress. 3, Opportunities to make independent decisions support child *autonomy*. Child chooses from a variety of music play activities, and each activity includes a wide range of materials. Activities include a wide range of materials and activity options so child can make choices for self and others. Therapist uses improvisational techniques to follow child-initiated changes in their music making (e.g., child changes tempo or style of playing). 4. Activities structured to support *caregiver-child interaction*. Activities are designed to structure and support reciprocal caregiver-child interactions. Therapist individualizes experiences to support increased frequency and quality of interactions.
Component 2: Music Play Resource Kit	Supports *independent use* of music play to manage distress between therapist-led sessions. Activities mirror content from therapist-led sessions. The kit includes: 1. Professional audio recording of music composed and/or arranged specifically for the AME intervention. 2. Age-appropriate musical instrument and play materials that correspond to each activity. 3. Activity cards designed to give children/caregivers information “at-a-glance” on ways they can use their kit.
Component 3: Session Planning & Caregiver Tip Sheets	1. Promotes caregiver *competence* about how children use play to cope and ways to engage their child in music play during transplant. 2. Promotes caregiver *autonomy* by empowering caregivers with skills/resources to support their child during treatment 3. Supports caregiver-child *relationships* through normalizing, music-based play activities

^*^ Table reprinted with permission in accordance with creative commons open access license ‘Attribution-Noncommercial 4.0 International’ (CC BY-NC 4.0) http://creativecommons.org/licenses/by-nc/4.0/ for the following publication: from Russ KA, Holochwost SJ, Perkins SM, et al. Cortisol as an acute stress biomarker in young hematopoietic cell transplant patients/caregivers: active music engagement protocol. *JACM*. 2020;26(5):424–434

#### Audio-storybooks (ASB) attention control condition

Trained music therapists also deliver the ASB condition, which was designed to control for attention from a provider, shared parent-child experiences, and audio-visual stimulation that comprise the non-therapeutic aspect of the AME condition. It offers parents/children opportunities to make choices and engage in an age-appropriate, non-music-based play activity. In each session, parents/children choose and listen to one of several illustrated children’s books with audio-recorded narration. In our prior work, this condition was acceptable to children and parents and did not demonstrate any significant benefits on our outcomes of interest, making it the best control condition for this trial [32–34].

### Treatment fidelity strategies

For this trial, our team developed treatment fidelity strategies based on our prior studies [57] and NIH Behavior Change consortium recommendations [58]. Specific strategies for the five specified fidelity components (design, provider training, treatment delivery, treatment receipt, and treatment enactment) are central to ensuring study rigor and reproducibility. A description of treatment fidelity strategies for this trial, including strategies to reduce risk for bias and contamination between conditions, are detailed in a separate publication [37].

### Outcome measures and data collection procedures

#### Parent (caregiver) report measures

Following informed consent, project managers at each site arrange a time for parents to complete baseline (T1) questionnaires during their first consolidation phase clinic appointment. A trained data collector administers questionnaires and remains available for questions. After completing T1 questionnaires, the project manager notifies the child/parent of their group assignment and schedules their first study condition session. Parents complete Time 2 questionnaires within 7 days of completing their last study session (session 4 for standard risk; session 8 for high risk).

The use of valid, reliable measures helps to ensure study rigor and reproducibility of results. All measures reflect careful consideration of psychometric properties, sensitivity to change, and response burden. Table 2 provides a list of measures, psychometrics, and administration schedule.

**Table 2:** Measures

Variable(s)	Measure	# Items	Reliability Evidence	Admin. Schedule	Done By
**Antecedent Factors**					
Demographics	Family Information Form	3	N/A	T1	Parent
Prior Distress w/ Hospitalization Parent Traumatic Stress Screener	Prior Illness Experiences Scale (PIES)[59] Abbreviated PTSD checklist (PCL-S)[60]	13 6	0.78† 0.94†	T1 T1	Parent Parent
Disease and Tx Characteristics	Diagnosis and Treatment Form	2	N/A	T2	PM/ Co-I
**Mediators**					
Child Stress Parent Stress	Salivary Cortisol Salivary Cortisol	N/A N/A	N/A N/A	Pre/Post Sessions 1–4	Data Collector
**Child Outcomes**					
Child Immune Function	Immunomodulatory Cytokines (Blood)*	N/A	N/A	Pre-Sessions 1, 4, 8	Nurse/ Phleboto-mist
Child Emotional Distress	CHQ – *Mental Health Subscale*[61]	16	0.81†	T1, T2	Parent
Child Quality of Life	KINDL^R^ [62]	20	0.89†	T1, T2	Parent
**Parent Outcomes**					
Parent Emotional and Traumatic Stress Symptoms	Profile of Mood States-Short Form (POMS)[63] Impact of Events Scale-Revised (IES-R)[64]	37 22	0.99** 0.84-0.91†	T1,T2 T1, T2	Parent Parent
Parent Quality of Life	Index of Well-being[65]	9	0.93†	T1, T2	Parent

†Cronbach’s alpha; **correlation with POMS

*Blood taken during scheduled draw

#### Biologic sample collections and storage during clinic visits

This study collects both saliva and blood. Both child and parent (caregiver) provide saliva for the detection of cortisol levels. Saliva collections occur before and after each intervention or attention control session at all 4 sessions for standard risk participants and the first 4 sessions for high-risk participants. The saliva is collected in a Salimetrics SalivaBio Oral Swab or SalivaBio Children’s Swab for the caregiver and child, respectively. The swab is immediately placed in the prelabeled storage tube, placed on dry ice post-collection, and stored at -80 C.

Blood is only collected from the children. At each site, 5 mL of blood is collected as part of routine study draws the morning of their sessions and used to isolate serum for cytokine analysis. This occurs at sessions 1 and 4 for all children with an additional draw at session 8 for high-risk children. Our primary site collects an additional 1 mL of blood to conduct a blood cortisol analysis for the purpose of monitoring adrenal function in a subset of the children on study. These collections occur at the child’s end of induction clinic appointment as well as sessions 1 through 4. Serum for cytokine analyses will be held until the end of the study at which point multiplexed analyses will be run. Serum for monitoring adrenal function in the children will be run on a yearly basis.

Treating physicians, at our three hospitals, have assigned clinic days and times (morning vs. afternoon). This will minimize variability in the timing of saliva collection between appointments. We will track day/time of collections and other factors that may contribute to child and parent cortisol interpretation (i.e., sleep, food/drink intake, medications).

#### Data collection on parent/child medications

We created a survey to identify and monitor sleep, food, and medication intake that may interfere with our ability to interpret cortisol collections. This survey is administered at the same time as our baseline participant questionnaires prior to any study sessions. The survey is structured in our database such that after parents fill out medication information for themselves and their children, they only need to update any changes that have occurred each visit for which there is a biologics collection. This process saves participants time and ensures smoother clinic flow.

#### Masking process

During study introduction and informed consent, the AME and ASB conditions are presented as equal conditions that inform our understanding about the use of play experiences, like music and stories, to reduce child and parent stress during cancer treatment. As such, child/parent dyads are masked to whether they are receiving the intervention or attention control condition. Data collectors administering baseline (T1) and post-intervention (T2) questionnaires are masked to participants’ group assignment. Should a data collector who is responsible for T2 data collection become aware of a participant’s group assignment, we assign another data collector unaware of group assignment to administer measures. Data collectors administering pre- and post-session saliva collections will not be masked to group assignment due to timing/proximity of collections to session delivery.

### Data analysis

#### Analysis of biomarkers

Saliva samples cortisol levels will be analyzed using manufacturer validated R&D® ELISA kits and controls. The levels of IL-1$\beta$, IL-6, TNF-$\alpha$, IFN-$\gamma$, IL-4, IL-10, and IL-13 in patient serum will be analyzed using a Bio-Rad multiplex immunoassay 7-plex kit and controls in the Multiplex Analysis Core. Reproducibility of these kits is validated by the manufacturer and each plate will be run with appropriate standards as well as internal controls to ensure reliability across lots. Kit lot numbers as well as certificates of analysis (provided by the manufacturer) will be kept with the study records.

#### Preliminary analyses

Prior to hypothesis testing, we will calculate coefficient alpha as a measure of internal consistency reliability on all multiple-item scales. Construct validity will be assessed by calculating Pearson or Spearman correlations among scales to determine if correlations are in the expected direction. We will present descriptive statistics for the AME group and the attention control group with respect to demographic and baseline outcome variables. We will control for child and parent age, site, risk level (standard or high), greater distress with prior hospitalizations, greater traumatic stress symptoms, time of collection of pre-session cortisol, and child and parent sex in all models. Age, site, and risk level are stratification variables. Time of collection of pre-session cortisol will account for differences based on standard diurnal differences. Although time of collection will be similar within each child, there will likely be between-child differences in time of collection (e.g., some children will have collections in morning and some in afternoon) that we will control for in analyses.

#### Missing data and multiple comparisons

We will compare all baseline variables between subjects who drop out of the study and those who do not using two-sample *t* tests, chi-square tests, or non-parametric equivalents as appropriate. MPlus software will incorporate participants who drop out before completion by using the MPLUS imputation method to perform a bias adjustment for missing data under maximum likelihood estimation and the assumption that data are missing at random. If we find that missing data are not missing at random, we will use a pattern mixture modeling approach to address; note we expect up to 25% attrition but minimal missing data on instruments (< 1%). We will adjust *p* value for multiple comparisons using the Bonferroni method for parent emotional/traumatic distress as there are two outcomes for this domain. For cytokine analyses, there are 7 cytokines of interest, so a Hochberg step-up approach will be used for these analyses.

#### Main analyses

For analyses below, we will analyze as randomized and attempt to collect outcome data on non-completers, following the intent-to-treat principle. For Aims 1 and 2, each outcome will be modeled separately, and we will examine sex as a biological variable by including sex as a covariate to control for its relationship with the outcomes and the interaction of sex and intervention group to examine if there are differential effects by intervention group. For Aim 1 (H1.1 and H1.2), the outcome will be the pre- to post- percent change in cortisol for each session. A linear mixed model will be fit with the independent fixed effects variables of intervention and session (1 to 4). Subject will be a random effect. We will use an F-test to test the intervention effect. For Aim 2 (H2.1, 2.2, 2.3), our primary goal is to test mediation effects. Mediation effects will be estimated in an ANCOVA setting, fitting the appropriate mediation models using MPlus [66] and then testing indirect effects using the percentile bootstrap approach to estimate the indirect effect [55]. The mediation model with two-path mediation effects specifies that the intervention will act through the mediator on the outcome and also have a direct effect on the outcome. Each outcome model will have 3 key predictors (intervention, two proximal mediators), and control for T1 outcome and covariates described above. For testing cortisol as a mediator, we will use (1) the estimated average percent change estimated from the mixed model, and (2) the % change from pre-session 1 to post session 4. In addition, we will look at child and parent cortisol levels both separately and together (multiple mediation model) in order to fully assess if/how they function together as mediators. Thus, for any single outcome, up to 6 models will be examined. For all mediation models in Aim 2, we will assess goodness-of-fit (GOF) using standard GOF measures (comparative fit index, root mean square error of approximation, and root mean square residual). For Aim 3, an exploratory aim, we will use graphical plots and non-linear repeated measures models to model the trend in percent changes in cortisol within and between intervention groups across the 4 sessions for all participants and across 8 sessions for high risk participants.

## Discussion of biomarker strategy

Increased HPA-axis activity stimulates the release and production of inflammatory biomarkers [15, 67, 68], which in turn is associated with negative health outcomes for individuals with cancer (diminished immune function) [14, 15] and their parents (traumatic stress symptoms) [69]. This evidence supports investigation of biological pathways underlying the use of active music to mitigate cancer-related stress. To measure these during active cancer treatment, there were three primary challenges to overcome in designing this trial.

The *first challenge* was to avoid phases of treatment involving glucocorticoid therapy (e.g., prednisone or dexamethasone which are synthetic analogs of cortisol) [70]. Treatment for ALL and TLyLy in the United States currently consists of 5 phases: induction, consolidation, interim maintenance, delayed intensification, and maintenance. Based on our team’s review of Children’s Oncology Group protocols for treating standard and high risk pediatric B-cell ALL, T-cell ALL, and TLyLy, glucocorticoids are not used during the consolidation and interim maintenance phases. Children with ALL/TLyLy receive 2–4 weeks of glucocorticoid therapy during induction. Induction lasts for 28 days and consolidation starts 1–2 weeks post induction so they will have been off glucocorticoid therapy for at least seven days [71, 72]. At least 80% of children with ALL/TLyLy do not show adrenal insufficiency as early as 7–14 days after stopping either prednisone or dexamethasone [70, 73]. The 20% who have continued adrenal insufficiency are easily identified via morning cortisol levels of < 3mcg/dl and symptoms that would delay the onset of consolidation (e.g., fever, hypotension, vomiting) [73]. Therefore, these data suggest that cortisol can be measured in this population during consolidation.

To further address concerns about possible adrenal insufficiency at the start of consolidation, we will examine blood cortisol levels in a sub-sample of study participants (Indianapolis site) at the end of induction therapy, and on days during consolidation therapy when participants are scheduled to receive study sessions 1–4. This will allow us to identify participants in the sub-sample with adrenal insufficiency at the start of consolidation, which will enable us to better interpret alterations in salivary cortisol levels over time in those individuals. It may also allow us to recognize patterns in salivary cortisol levels that would indicate adrenal insufficiency to identify and control for participants at other sites with this same issue. The addition of the child blood cortisol collections will increase study rigor and interpretation of child salivary cortisol.

The *second challenge* was to select appropriate timing for cortisol collection while identifying strategies to avoid burdening children and parents [18, 19]. Cancer treatments’ interference with sleep schedules can disrupt diurnal rhythms of cortisol production [74]. To address these challenges, we will examine more immediate changes in cortisol that occur around the time of the AME or attention control experience (pre/post-session) and over time (across 4 weekly sessions); an approach taken in three previous pediatric studies [75–77]. This also mitigates the potential for increased burden on parents associated with collecting, storing, and transporting multiple samples from home to clinic, and related concern about sample viability. Over time, we expect that the size of within-child percent decrease in cortisol among AME children will become larger than the corresponding decrease observed in attention control children. Focusing on relative levels of cortisol within children minimizes the influence of children’s absolute levels of cortisol, for which the diurnal rhythms may be altered. With regard to timing, treating physicians at our three hospitals have assigned clinic days and times (morning vs. afternoon), and this will help minimize variability in the timing of salivary cortisol collection between appointments (e.g., patient A is always in clinic in the morning, patient B is always in clinic in the afternoon). In addition, we will statistically control for timing of cortisol collection as necessary.

The *third challenge* was to select and use highly sensitive measures of immune function. The capacity to modulate immune function in response to stress is fundamentally adaptive [14, 78–80], but chronic or severe stress can dysregulate the immune response, including the function of cytokines that act as messengers to the immune function cells [79]. Intensity and duration of stress can have significant negative effects on immune cell distribution and function via increases in glucocorticoids (e.g., cortisol). Effective immuno-protection requires that leukocytes rapidly respond to sites of infection or other potential risk (such as a wound or surgical site) [80]. This ability, in the face of short-term stress, is a necessary and underappreciated function of stress and stress hormones. However, in the long term, these same responses can lead to immune-pathology and decrease the child’s resistance to infection, wound healing, and even the cancer treatment itself [79]. The immune markers to be utilized in our study were chosen to capture information on the signaling occurring within the immune system that will modulate the function and phenotypes of immune cells and ultimately affect immune function.

Our team has worked to anticipate and address challenges associated with studying the interrelated stress of young children with cancer and parents during cancer treatment to create a rigorous trial design. However, as with any clinical trial, we recognize that a variety of factors such as the nature of the disease, unexpected drug interactions, and unanticipated treatment deviations will occur. This includes development of rigorous treatment fidelity strategies and the formation of interdisciplinary teams that remain engaged over the life of the trial - monitoring and assessing unexpected situations and making quick decisions to account for variations and maintain study integrity.

As one of the first pediatric music intervention studies to examine biomarkers of stress and immune function, findings from this trial will inform clinical practice in important ways including improved understanding about how active music effects parent and child stress (and the interrelated nature of their stress) at the biological level, and the potential benefit and clinical utility of active music to improve immune function in children during cancer treatment.

## Electronic supplementary material

Below is the link to the electronic supplementary material.

Supplementary Material 1

## Data Availability

Not applicable.

## Declarations

### Protocol version

Issue Date: 26 October 2022; Protocol Amendment Number: A033; Version 13.

## List of abbreviations

ALL: Acute Lymphoblastic Leukemia
AME: Active Music Engagement
ASB: Audio-Storybooks
CSM-MT: Contextual Support Model of Music Therapy
GOF: goodness-of-fit
HPA: Hypothalamic-Pituitary-Adrenal
TLyLy: T-cell Lymphoblastic Leukemia
T1: Time 1 (baseline)
T2: Time 2
